# Peptide-equipped tobacco mosaic virus templates for selective and controllable biomineral deposition

**DOI:** 10.3762/bjnano.6.145

**Published:** 2015-06-25

**Authors:** Klara Altintoprak, Axel Seidenstücker, Alexander Welle, Sabine Eiben, Petia Atanasova, Nina Stitz, Alfred Plettl, Joachim Bill, Hartmut Gliemann, Holger Jeske, Dirk Rothenstein, Fania Geiger, Christina Wege

**Affiliations:** 1 Department of Molecular Biology and Plant Virology, Institute of Biomaterials and Biomolecular Systems, University of Stuttgart, Pfaffenwaldring 57, 70569 Stuttgart, Germany; 2 Institute of Solid State Physics, University of Ulm, Albert-Einstein-Allee 11, 89081 Ulm, Germany; 3 Karlsruhe Nano Micro Facility (KNMF), Karlsruhe Institute of Technology, Hermann-von-Helmholtz-Platz 1, 76344 Eggenstein-Leopoldshafen, Germany; 4 Institute of Functional Interfaces (IFG), Karlsruhe Institute of Technology, Hermann-von-Helmholtz-Platz 1, 76344 Eggenstein-Leopoldshafen, Germany; 5 Institute for Materials Science, University of Stuttgart, Heisenbergstraße 3, 70569 Stuttgart, Germany

**Keywords:** biomineralization, charge-relay system, peptide, silica, tobacco mosaic virus (TMV)

## Abstract

The coating of regular-shaped, readily available nanorod biotemplates with inorganic compounds has attracted increasing interest during recent years. The goal is an effective, bioinspired fabrication of fiber-reinforced composites and robust, miniaturized technical devices. Major challenges in the synthesis of applicable mineralized nanorods lie in selectivity and adjustability of the inorganic material deposited on the biological, rod-shaped backbones, with respect to thickness and surface profile of the resulting coating, as well as the avoidance of aggregation into extended superstructures. Nanotubular tobacco mosaic virus (TMV) templates have proved particularly suitable towards this goal: Their multivalent protein coating can be modified by high-surface-density conjugation of peptides, inducing and governing silica deposition from precursor solutions in vitro. In this study, TMV has been equipped with mineralization-directing peptides designed to yield silica coatings in a reliable and predictable manner via precipitation from tetraethoxysilane (TEOS) precursors. Three peptide groups were compared regarding their influence on silica polymerization: (i) two peptide variants with alternating basic and acidic residues, i.e. lysine–aspartic acid (KD)*_x_* motifs expected to act as charge-relay systems promoting TEOS hydrolysis and silica polymerization; (ii) a tetrahistidine-exposing polypeptide (CA_4_H_4_) known to induce silicification due to the positive charge of its clustered imidazole side chains; and (iii) two peptides with high ZnO binding affinity. Differential effects on the mineralization of the TMV surface were demonstrated, where a (KD)*_x_* charge-relay peptide (designed in this study) led to the most reproducible and selective silica deposition. A homogenous coating of the biotemplate and tight control of shell thickness were achieved.

## Introduction

Amorphous silica (SiO_2_) precipitated from silicate precursor sols comprises a wide range of versatile materials applied in various technological approaches, for example, as a structural modifier or filler in rubber [1], food [2–3] or healthcare products [4], bioceramics for medical purposes [5], mesoporous nanoparticulate or tubular drug delivery systems as reviewed in [6], sensor surfaces [7], or biocatalytic formulations as reviewed in [8]. An important focus of research and industry lies on the development of nanoscale materials, enabling the further miniaturization of devices and effector units, in addition to a reduced consumption of resources. In the field of functional mineral synthesis, significant progress has been made in using nanodimensional biological templates, allowing specific coating with inorganic materials to yield hybrid particles of predetermined structure and composition [9–11]. The surfaces of optimal templates nucleate and direct the formation of inorganic materials from suitable precursors, resembling a natural matrix-mediated mineral deposition in living organisms known also as “biologically controlled mineralization” [12–13]. Such bio-inspired mineralization approaches can accomplish precise coating processes and offer several benefits such as environmentally friendly fabrication routes and reaction parameters compatible with biological structures, namely low synthesis temperature and aqueous deposition media. In this context, tube- or rod-like templates of high aspect ratio are of particular interest, since they enable the fabrication of elongated nanostructures, which are otherwise difficult to obtain. This is because chemical synthesis or technical approaches applied at mild conditions commonly generate spherical structures [14]. Mineral nanofibers of predetermined size are of major importance for the preparation of functional films and extended 3D materials. Hence, anisotropic scaffolds such as high molecular weight polymers [15], carbon nanotubes [16], peptide nanotubes [17], certain plant viruses [18–21], filamentous bacteriophages [22–23], and bacterial flagellae [24] have been evaluated for their applicability on a technical scale. To achieve control over mineral precipitation, the modification of the template by chemical conjugation of peptides [16], poly(ethylene glycol) (PEG) [22], aniline [25–26], or succinamate [27] has been reported.

Virus-based templates have gained especially important roles in the synthesis of organic–inorganic hybrid nanostructures. They combine several advantages, namely high availability, robustness and an exact replication of the particle shape and dimension, which are genetically determined and result in a narrow size distribution. Different species such as the fibrous bacteriophage M13, icosahedral cowpea mosaic virus (CPMV), or tubular tobacco mosaic virus (TMV) were used as templates for coating with inorganic materials including Pt, Au [28], Ag [29–30], Pd [31–32], TiO_2_ [33], SiO_2_ [34], NiO [35], CdS [21], CoPt, FePt, ZnS [27,36] and ZnO [37–39]. Among the virus-based templates, plant viruses are especially suitable nanostructured scaffolds because of their biological safety for humans, animals, and their commensal bacteria. TMV is a widespread plant-infecting pathogen, which can be isolated in large amounts from susceptible plants [40]. TMV particles are highly ordered, supramolecular complexes, consisting of a single-stranded helical RNA and ≈2130 identical coat protein (CP) subunits arranged around the RNA molecule, which is completely buried inside the protein shell [41–46]. The viral particle has an average length of 300 nm and an outer and inner (channel) diameter of 18 nm and 4 nm, respectively. TMV has become a powerful building block in bionanotechnology due to its tube-like structure, high stability under a wide range of different conditions (e.g., pH, temperature, solvent), low production costs and multivalent CP surface [18,47–48].

The CP subunits of TMV can be genetically or chemically modified for the presentation of effector molecules [35,49–52]. Modified TMV templates maintain their 3D structure along with preserved particle stability, which is a prerequisite for the subsequent mineralization of inorganic materials. Furthermore, the length and also the overall shape of TMV-derived particles can be altered by means of engineered, non-natural RNA molecules, supporting the assembly of artificial, non-infectious, TMV-like nucleoprotein tube systems. This technology was even refined to allow the production of kinked boomerang, branched tetrapod and multiarmed nanostar structures [53–54], or into particles fashioned evenly with mixtures of two or more functional groups at predefined ratios [51].

To vary and control the deposition of inorganic minerals on TMV templates, extensive modifications of the surface amino acids are desirable. They enable defined alterations of the outer TMV–CP surface charge and the introduction of specific amino acid motifs, guiding the nucleation and growth of mineral coatings around the TMV core. This is in analogy to natural biomineralization-directing protein domains identified for various organisms [55–59]. Direct genetic modification of the TMV–CP sequence is, however, limited in view of the extent of alteration tolerated by virus particles upon their multiplication in plants, regarding number and composition of exchanged or inserted amino acids. In addition, high-throughput screening of different surface-expressed peptides is restricted upon TMV “farming” due to the required 10–14 days for TMV mutant accumulation. Bacterially expressed CP can be engineered to a much higher extent and integrated into TMV-like particles reconstituted in vitro in substantial amounts [51]. However, purification of such protein types from the bacteria cultures is much less efficient compared to CP isolation from intact TMV particles from leaf tissues.

**Figure 1:**
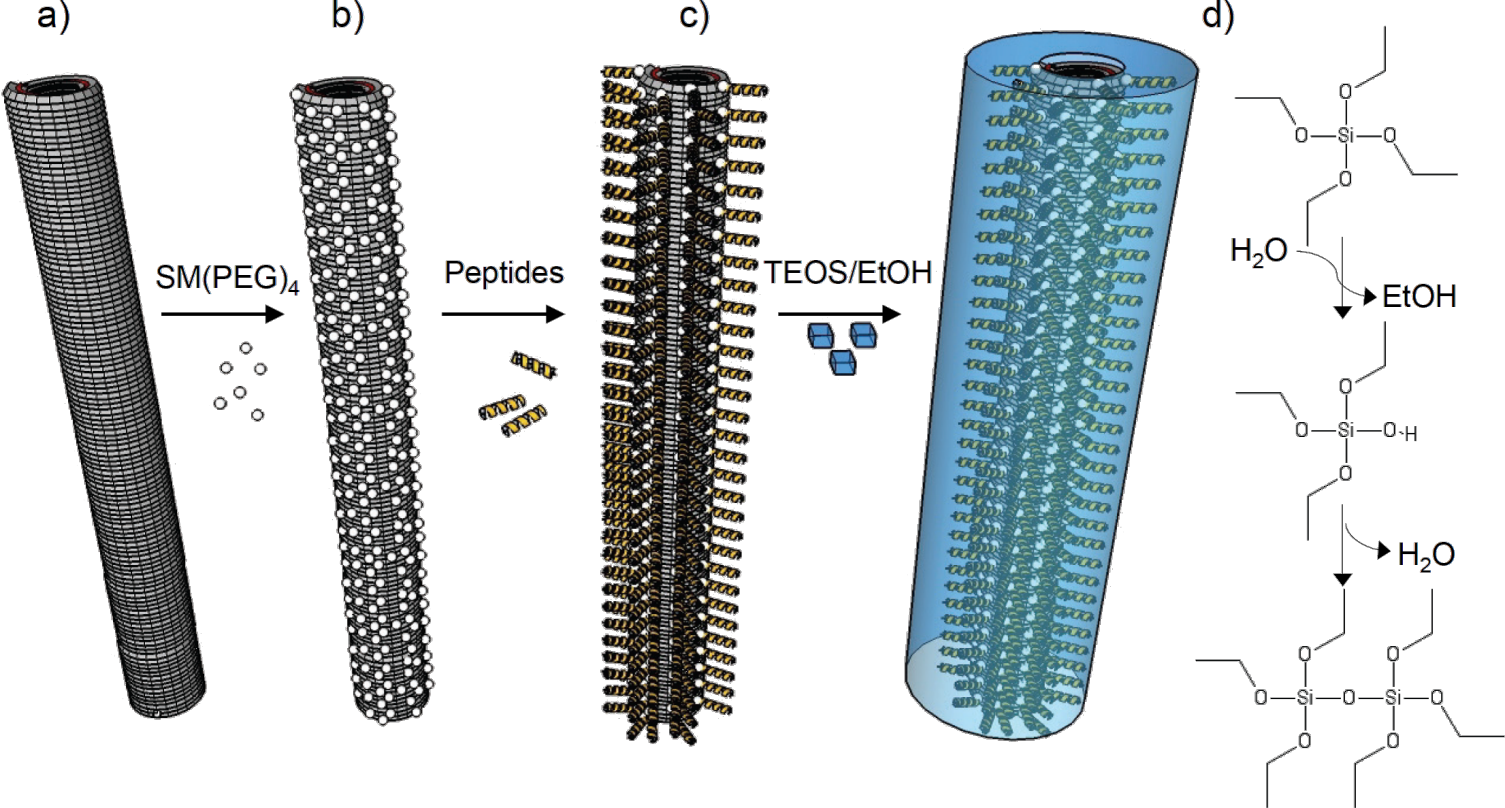
Schematic representation of the chemical modification and mineralization of tobacco mosaic virus (TMV) nucleoprotein nanotubes. (a) Genetically engineered virus particles with thousands of surface-exposed amino groups of lysine residues (TMV_Lys_) served as biotemplates for chemical conjugation reactions. (b) Hetero-bifunctional linker molecules (succinimidyl-(*N*-maleimidopropionamido) ester, SM(PEG)_4_) were coupled to TMV_Lys_ via *N*-hydroxysuccinimide (NHS) ester-mediated crosslinking with lysine primary amines, yielding amide bonds. (c) Mineralization-affecting peptides were conjugated to the maleimide-activated SM(PEG)_4_ linker portion via the sulfhydryl groups of their terminal cysteine residues, yielding stable thioether linkages. The resulting functionalized TMV templates fashioned with a dense peptide coating were (d) subjected to silica mineralization via hydrolysis and condensation of a tetraethoxysilane (TEOS) precursor in solution (mechanism indicated).

Therefore, we have followed a third strategy and made use of plant-enriched, moderately engineered TMV templates, exposing selectively addressable reactive surface groups. These were subjected to chemical conjugation of synthetic peptides meant to regulate subsequent coating with silica (workflow indicated in Figure 1). This procedure is insensitive to both size and sequence of the peptide of choice, and the generation of various types of decorated TMV rods is fast. The amino acid sequences employed had been previously delimited by phage display to affect mineralization in our work [60] or by other researchers [17], or were predicted to influence silica deposition based on the literature [61]. Control experiments were carried out in parallel with bare TMV equally treated, in order to assess its capacity for silica nucleation in the absence of additional peptide domains.

Generally, it still remains a challenge to predictably, selectively and uniformly coat individual nanotemplate particles with silica. For this purpose, sol–gel condensation from precursors in alcoholic solutions seems most viable according to the conditions established by Stöber et al. [62] for the fabrication of plain silica spheres. Reaction parameters such as time, temperature, pH, solvent composition and precursor, as well as catalyst concentration, affect the thickness of the mineral coating, in interdependence with the chemistry and charge distribution of the surface of the core [22,26–27,63–64]. During the mineralization process, great effort is needed to avoid non-templated byproducts, as well as aggregation, re-organization and precipitation of the templates into extended superstructures of amorphous silica-template composites.

Several earlier studies have demonstrated that native TMV capsids are effective biological nucleation cores for the deposition of mineral layers from silicate sols on their surfaces. This is typically carried out via hydrolysis and condensation of tetraethoxysilane (also known as tetraethylorthosilicate, TEOS) in alcohol-containing media (see Figure 1 for the mechanistic scheme). Obviously, the viral coating (which exhibits patches of both positively and negatively charged amino acids in nanometric vicinity to each other) is prone to silica deposition by itself. Concomitant with silicification reactions, however, TMV particles presented extensive rearrangement into head-to-tail structures [34,65]. These were laterally aligned or even star-shaped [64] 3D arrays in many of the studies, reflecting and expanding the well-known capacities of these bio-nanorods to form various aggregates up to liquid–crystalline phases. In contrast, protocols resulting in nanoparticulate TMV–silica composites devoid of inorganic background granules and, moreover, with closed shells of well-adjustable thickness, are a matter of intense investigation. Such methods would enable the manufacture of novel TMV derivatives, further expanding their application potential to miniaturized arrays and devices [66–68] and “smart” functional materials [31,69] for numerous purposes. TMV–inorganic hybrids will likely yield rigid and durable [70] technical components, which are also compatible with biological molecules and activities [71].

To fabricate thick, continuous silica coatings (e.g., on immobilized TMV rods), polyaniline interlayers were employed to shield both charges and hydrophobic patches of the viral protein coating before subjecting it to TEOS-mediated mineralization [26]. A more refined and tighter control of the silica mineralization can be achieved by ordered, repetitive arrangements of differently charged protein domains and specific functional groups therein. This was demonstrated in vitro by help of various mineralization-inducing peptides [55–56,61,72]. Positively charged amino acid residues (Lys, Arg) in such peptides electrostatically interact with siloxane groups, while Ser, His and Asp may undergo hydrogen bonding or polar interactions with solute or nanosized colloidal mineral precursors [7,60,63,72–74]. Additionally, negatively charged amino acids that are present are supposed to have an enhancing effect on TEOS hydrolysis. This is especially true if they are closely adjacent to positive charges, where such combinations may act as charge-relay systems [61,75]. Effective peptides may resemble motifs found in natural silica biomineralization-directing proteins (in both their amino acid composition and sequence [58]), but may also comprise randomly assembled sequences resulting from mere in vitro library screening.

Since a growing body of experience with relatively diverse silicification-guiding peptides is available, we decided to install a number of distinct amino acid sequence types on structure-directing TMV nanorods. This allows for the systematic investigation of their influence on silica coating reactions via ethanol-containing TEOS on this viral backbone for the first time.

Genetically modified TMV particles (TMV_Lys_) with an accessible amino group on every CP subunit [76] were chemically equipped with a dense peptide coating via succinimidyl ester-activated, bifunctional, PEG-based linkers, and their subsequent maleimide-mediated conjugation to thiol groups of terminal cysteine residues present in every peptide. Five different peptide sequences were selected (see Table 1): (i) (KD)_5_C and (KD)_10_C with alternating amino and carboxyl functionalities (sequences KDKDKDKDKDC and KDKDKDKDKDKDKDKDKDKDC, respectively) on the basis of Kuno et al. [61]; (ii) CA_4_H_4_ (sequence CAAAAHHHH) according to Yuwono and Hartgerink [17], with two stretches of different amino acid residues arranged blockwise to expose a cluster of imidazole side chains; and (iii) 44C (HSSHHQPKGTNPC) and 31C (HHGHSPTSPQVRC), two ZnO-binding peptides isolated by phage display [60]. The distinct peptide-fashioned TMV_Lys_ templates were incubated in TEOS precursor solution in parallel with linker-coated and plain TMV_Lys_ controls (and in some tests wildtype TMV_wt_) under equal conditions. The products were analyzed and compared to determine favorable TMV template–peptide combinations for specific silica mineralization.

**Table 1:** Mineralization-affecting peptides installed on TMV templates to compare their influence on silica deposition from TEOS. Amino acid sequence and total number (aa) are indicated for each peptide. Molecular weight (*M*_w_), isoelectric point (pI), and net charge at pH 8.0 and 5.5 were calculated with Protein Calculator v3.4 [77].

Name	Abbreviation	Sequence	aa	*M*_w_ (g/mol)	pI	Net charge at pH	
						8.0	5.5

(KD)_5_C	KD5	KDKDKDKDKDC	11	1337.5	6.25	−0.8	0.4
(KD)_10_C	KD10	(KDKDKDKDKD)_2_C	21	2553.8	6.62	−0.8	0.7
CA_4_H_4_	AH	CAAAAHHHH	9	945.0	7.52	−0.6	3.6
44C	44C	HSSHHQPKGTNPC	13	1429.5	8.31	0.3	3.7
31C	31C	HHGHSPTSPQVRC	13	1442.6	8.31	0.4	3.7

## Results and Discussion

### Surface functionalization of TMV_Lys_ templates by conjugation of mineralization-promoting peptides

**Figure 2:**
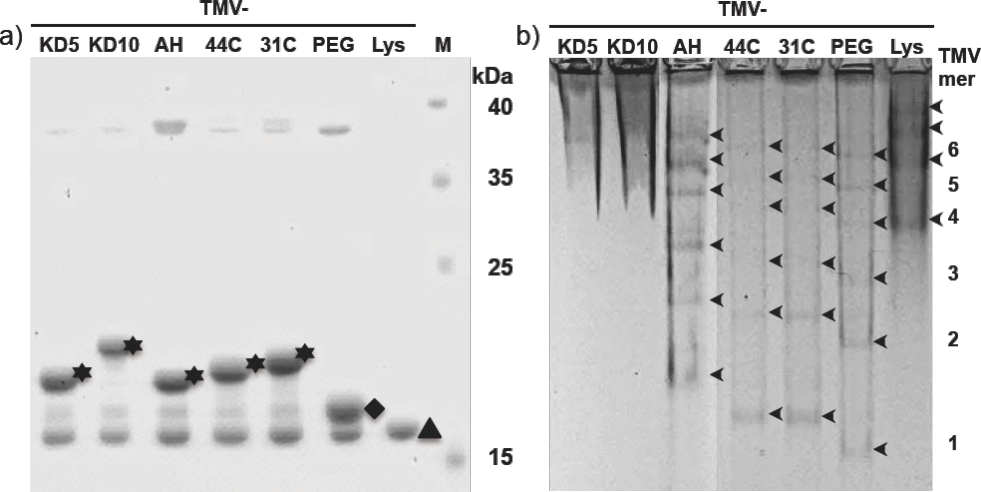
Gel electrophoretic analysis of chemically modified TMV–Lys particles. (a) SDS-PAGE shows retarded bands of CPs modified with the linker SM(PEG)_4_ (diamond, PEG), or after coupling SM(PEG)_4_ and different peptides (stars, peptides as indicated above), compared to unmodified CP_Lys_ (triangle, Lys). (b) Peptide-equipped TMV–Lys particles exhibiting different separation patterns during native agarose gel electrophoresis, indicating various states of head-to-tail aggregation in combination with distinct negative overall charges. Moieties exposed on the TMV templates are indicated (abbreviations as in Table 2). Numbers on the right: approximate numbers of TMV particles in head-to-tail aggregates (in relation to lane “TMV–PEG”).

To nucleate and govern the deposition of silica shells, functionalized plant viral nanorod templates were generated by linker-assisted chemical conjugation of mineralization-active peptides to the outer surface of genetically modified TMV_Lys_ particles from plants. Every CP_Lys_ subunit provided a primary amine group of a lysine residue at the protein's C terminus. This resulted in ≈2130 sites selectively accessible to NHS ester-mediated coupling reactions per rod [76]. These were equipped with hetero-bifunctional crosslinker molecules (succinimidyl-(*N*-maleimidopropionamido)-tetraethylene glycol ester, SM(PEG)_4_) serving as spacers and adapters for mineralization-affecting peptides. These were installed via maleimide-mediated conjugation of the cysteine sulfhydryl groups of the peptides. The resulting five distinct types of TMV_Lys_–PEG–peptide particles with their different CP derivatives are listed in Table 2, as well as the linker-fashioned and plain TMV_Lys_ templates used as references. The abbreviation scheme used was the following: abbreviations underscore the relevant functionalities or amino acids exposed; therefore, TMV_Lys_ is named TMV–Lys from now on. Covalent conjugation of peptides was confirmed for both single CPs and intact TMV particles by denaturing and native gel electrophoresis, respectively. Peptide modification of CPs resulted in a band shift with respect to increasing molecular weight, as compared to nonmodified CP in denaturing sodium dodecyl sulphate polyacrylamide gel electrophoresis (SDS-PAGE) (Figure 2a). The efficiency of peptide conjugation was determined by the ratio of the band intensities of modified and nonmodified CPs after Coomassie Blue staining. The binding efficiencies to individual CP subunits were ≈60% for all investigated peptides, corresponding to about 1250 peptides exposed on every 300 nm rod. The molecular weights of the differently modified CPs were in good agreement with the values calculated for the distinct conjugates (Table 2).

**Table 2:** Composition of TMV derivatives used in this study. Calculated and measured molecular weight (*M*_w_) of modified CP species were in good agreement.

TMV derivative	Abbreviation (TMV–)	Composition of TMV derivative			Calculated *M*_w_ of CP conjugate (kDa)	Measured *M*_w_ of CP conjugate^a^ (kDa)
		TMV_Lys_	SM(PEG)_4_	Peptide		

TMV_Lys_–PEG–(KD)_5_C	KD5	+	+	(KD)_5_C	19.5	19.9
TMV_Lys_–PEG–(KD)_10_C	KD10	+	+	(KD)_10_C	20.7	21.9
TMV_Lys_–PEG–CA_4_H_4_	AH	+	+	CA_4_H_4_	19.1	20.2
TMV_Lys_–PEG–44C	44C	+	+	44C	19.6	20.4
TMV_Lys_–PEG–31C	31C	+	+	31C	19.6	20.9
TMV_Lys_–PEG	PEG	+	+	–	18.1	18.2
TMV_Lys_	Lys	+	−	–	17.6	17.4

^a^Measured *M*_w_ values are derived from SDS-PAGE band analyses via retardation factor values determined by ImageJ software [78] and calibration curves obtained from *M*_w_ standards separated on the same gel.

The intact TMV particles were analyzed by native gel electrophoresis (0.9% agarose in TBE buffer, pH 8.0; Figure 2b). The linker coating of the control derivative TMV_Lys_–PEG (TMV–PEG) exhibited an increase in negative net charge in comparison to TMV–Lys, resulting in a higher electrophoretic mobility. This effect was reduced by the conjugation of mineralization-affecting peptides: TMV_Lys_–PEG–CA_4_H_4_ (TMV–AH), TMV_Lys_–PEG–44C (TMV–44C) and TMV_Lys_–PEG–31C (TMV–31C) exhibited retarded bands, which indicated the linkage of the peptides to the TMV–PEG template. TMV derivatives TMV_Lys_–PEG–(KD)_5_C (TMV–KD5) and TMV_Lys_–PEG–(KD)_10_C (TMV–KD10) could not be separated under the conditions applied: both samples did not migrate into the gel phase to a sufficient extent.

### Zeta potential measurement

**Figure 3:**
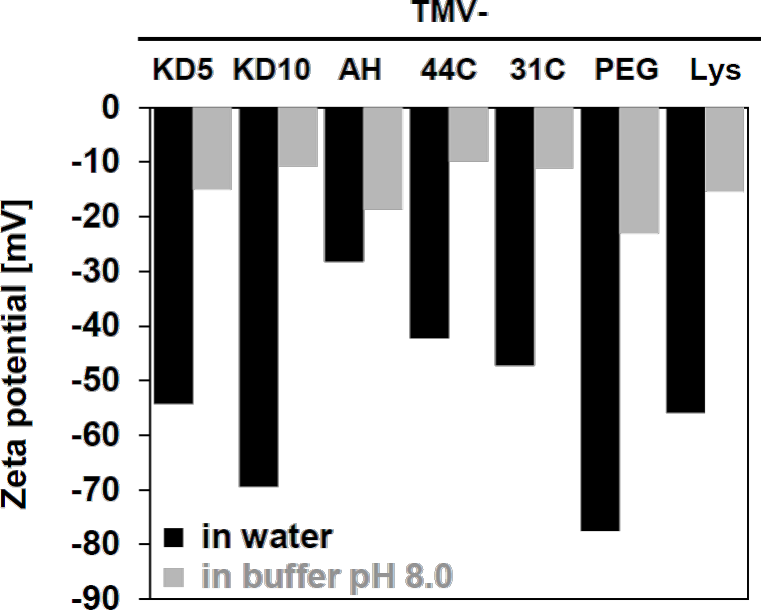
Zeta potential of bare and chemically modified TMV–Lys particles in ddH_2_O or 30 mM Tris-HCl pH 8.0, respectively (modifications of TMV rods indicated above).

The zeta potentials (ZPs) of TMV**–**Lys nanorods and their derivatives were determined using a Malvern NanoSizer at a virus particle concentration of 0.5 mg/mL in ultrapure water (ddH_2_O) and in 30 mM Tris-HCl buffer, pH 8.0, respectively (Figure 3). The ZPs measured in ddH_2_O were in general more negative (−28 mV to −78 mV) compared to those determined in buffer (−10 mV to −25 mV), owing to the lower pH of ≈5.5 of ultrapure water with CO_2_ dissolved in equilibrium with that in the air [79]. In addition, the increased electrolyte concentrations in the buffer lead to an enrichment of counter ions in the proximity of the TMV nanorods and thus a steeper decrease of the potential within a shorter distance from their surface (decrease of the Debye length). Therefore, ZP values measured in ddH_2_O are closer to the electric surface (Stern) potential of the particles [80].

The ZPs of the TMV derivatives (Figure 3) were correlated with the calculated isoelectric point (pI) of the conjugated peptides (see Table 1), and in most cases with the effective particle charges affecting their relative mobility in native agarose gel electrophoresis (see Figure 2b). Peptides 44C and 31C both are predicted to exhibit moderate positive charges (of about 0.3 or 0.4, respectively) at pH 8.0, while peptides AH (−0.6), KD5 and KD10 (both −0.8) are supposed to be negative. As coating of the TMV particles with maleimide-reactive SM(PEG)_4_ linker molecules devoid of peptides (TMV–PEG) introduced the most negative net charge (Figure 2b), all peptide-fashioned TMV derivatives had less negative ZPs compared to the linker-modified control (with about −80 mV in water and about −25 mV in buffer). While the ZP values of four products were largely in line with the calculated charges of the peptides (with TMV–44C and TMV–31C shifted to significantly less negative ZP values compared to those of TMV–KD5 and –KD10), the absolute ZP determined for TMV–AH was shifted most extensively to more positive values, due to the contribution of the uncharged alanines (Figure 3). This reflects the sheath of tetrahistidine clusters exposed by the C-termini of peptide AH. Bare TMV–Lys templates with their plain protein coating exhibited ZP values close to those of TMV–KD5.

At high concentrations, TMV–AH aggregated into bundle-like structures in water but not in buffer. Such agglomerates could be separated by ultrasound; however, re-aggregation occurred after short time. 44C- or 31C-functionalized TMV formed raft-like aggregates in both water and buffer (as detected also after their mineralization, see SEM analysis below).

For inorganic particles, the physical stability of dispersions increases with the magnitude of the ZP. That is, highly negative or highly positive ZPs typically both result in stable suspensions [81–82] due to Coulomb repulsion. The organic TMV template structures thus behaved analogously, with the agglomerating species TMV–AH, –44C, and –31C exhibiting the lowest ZP magnitudes in water. TMV–Lys with an absolute ZP value above 55 did not show aggregation at all.

### Mineralization of functionalized TMV templates

The different TMV templates were subjected to silica deposition by dispersion in a buffer-free deposition solution of ≈11% (v/v) TEOS precursor solution in ≈45% (v/v) ethanol in ultrapure water (resulting in a pH of ≈5.5) under agitation (500 rpm) at 25 °C for up to twelve days in parallel experiments (see Experimental section). These conditions were adapted with respect to the ethanol concentration from an earlier comparative study on the mineralization capacities of distinct kinds of peptides [61]. The method was established in initial tests to achieve improved control over mineralization kinetics and product characteristics with peptide-equipped TMV templates. This is in comparison to protocols used for the TEOS-mediated silicification of bare [21,34,64–65,83] or aniline-coated [26] TMV. Those protocols all employed reaction mixes of either alkaline or significantly lower pH (in most cases in buffer-free solutions) in variable ethanol concentrations and in one study supplemented by (3-aminopropyl)triethoxysilane (APTES) [64].

**Figure 4:**
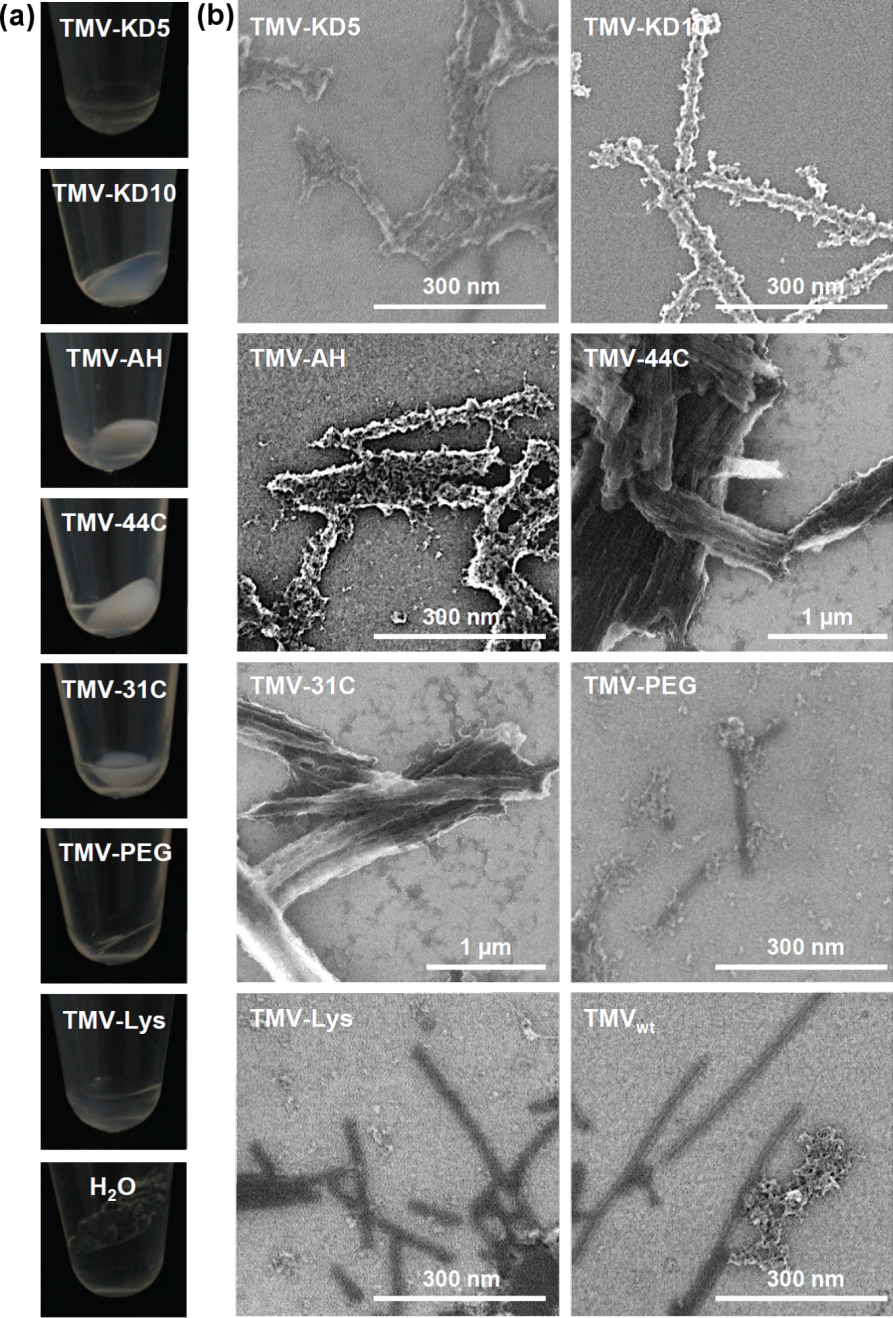
SiO_2_ deposition reactions using functionalized and non-modified TMV templates, as indicated. (a) Images of sedimented products, and (b) corresponding SEM analysis. TMV–Lys-template (or water control) solutions were mixed with absolute EtOH (99.9%) and TEOS in a 4:4:1 volume ratio. Reaction products were sedimented by centrifugation (after 7 days of incubation in (a) or 10 days in (b)), resuspended in ddH_2_O and prepared for SEM (for details, refer to text).

All TMV templates with absolute ZP magnitudes above 50 mV showed a good dispersion in the mineralization solution, while TMV–AH, –44C and –31C did not form stable suspensions. At different reaction times, products were collected by centrifugation. After seven days of incubation, inorganic material sedimented from all reaction mixes, regardless of the presence or absence of TMV templates (Figure 4a). The precipitates were transparent in the presence of TMV–KD5, –PEG, and –Lys, as well as for the reference sample without template, whereas the sediments of TMV–KD10, –AH, –44C and –31C appeared milky white. In the absence of TMV, the reaction solution completely solidified, while all suspensions containing TMV templates remained liquid during the course of silica condensation (Figure 4a).

The morphology of TMV hybrid products subjected to mineralization for ten days was analyzed by transmission electron microscopy (TEM; see below, data not shown), and high resolution scanning electron microscopy (HRSEM; Figure 4b). A clear difference in the mineralization of functionalized and non-functionalized TMV templates was observed: Only nanorods presenting the silica-binding peptides KD5 or KD10 showed continuous inorganic surface coatings on every single particle, with no significant agglomeration of the virus hybrids. Furthermore, deposition on these templates was highly specific; only a low amount of non-bound silica particles had formed (Figure 4b). The templates TMV–AH, –31C, and –44C induced silica deposition as well, but in contrast to TMV–KD5 and –KD10, the resulting composites did not contain separate rods anymore, but rather formed extended aggregates and bundles up to the µm size regime, especially pronounced for –31C and –44C. TMV templates lacking specific effector peptides (i.e., TMV–PEG, TMV–Lys, TMV_wt_) did not show any substantial inorganic coating at all, instead, some unspecific silica precipitation was observed (Figure 4b). It is known for in vitro systems that an alternating arrangement of lysine and aspartic acid residues (as in peptides KD5 and KD10) enhances dehydration of the TEOS precursor in the mineralization solution, in direct comparison to blockwise arrangements of the same amino acids. This results from an increased number of active sites for charge-relay effects [61]. The results of our comparative tests showing superior capacities of KD5 and KD10 to induce local silicification are in line with these earlier observations. This illustrates the beneficial effect of amino acid-based charge relay on a spatially directed TEOS conversion. Silica precipitation by sol–gel reaction from precursor solutions is likely to involve a gradual growth of individual silica nucleation cores rather than single or few specific phase transformations [74]. Hence, high surface densities of cooperating starter sites (such as repetitive KD pairs) may provide the best chance for an even growth of mineral shells, which are induced simultaneously at numerous closely adjacent sites.

The other peptides explored in this study, AH, 31C and 44C, all contain histidine residues as potential mineralization effectors. Their imidazole rings can catalyze hydrolysis of the TEOS precursor, resulting in deprotonated, negatively charged silicic acid, which then accumulates in the vicinity of the positively charged amino acids to facilitate silica mineralization [17]. The good efficiency of AH in promoting silica sheath formation from TEOS in the context of amphiphilic peptide fibers has been demonstrated [17]. 31C and 44C had not been tested with TEOS before, as they were originally identified due to their ZnO binding properties (data not shown) [60]. The agglomeration and bundle formation we found for all three respective mineralized TMV templates might be due to their aggregation before the mineralization process, as it is known for histidine-presenting TMV particles [51,84–85]. This is also indicated by their absolute ZP values of <50 mV measured in this study.

Most of the silicification-active peptides that convey the dehydration of precursor molecules such as TEOS [55–56,73,86–87] contain disproportionate amounts of positively charged amino acid residues (lysine, arginine or histidine). This reflects the design of silaffin, a natural silica-mineralizing protein rich in lysine and arginine residues [58,88]. Therefore, we speculated that bare TMV–Lys templates could also support the formation of silica shells in TEOS solution. The effect could be greater since the viral CPs are known to be N-terminally acetylated [89] and thereby might act as repetitive charge-relay systems on the viral surface. However, we could not detect any silica coating on TMV–Lys templates under the conditions applied. This may be due to the surrounding amino acids in the CP environment, which might slow down or even inhibit putative mineralization-supporting activity of the lysine moiety of the CP.

TMV_wt_ was also not mineralized in this experimental setup to an electron-optically detectable extent. This is in contrast to the strong and much faster mineralization of TMV_wt_ particles from TEOS solution in alkaline or more acidic pH regimes, as performed in other labs and described above.

**Figure 5:**
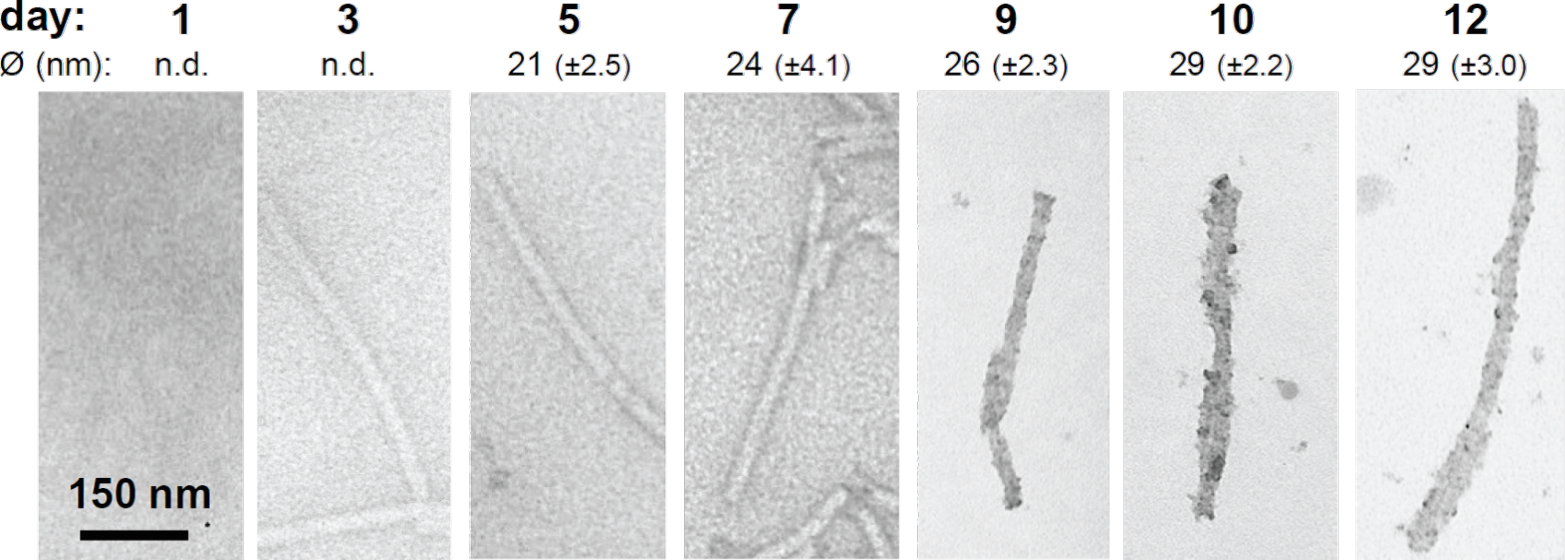
Time-resolved monitoring of silica shell growth on TMV–KD10 templates: TEM analysis of non-stained specimens, after the reaction times indicated above. Total average diameters (Ø ± standard deviations) of mineralized TMV–KD10-hybrids were determined from 11–15 randomly selected nanorod products collected between one and twelve days of incubation.

TEM analyses of the mineralized products confirmed the findings for the distinct TMV templates (not shown), with TMV–KD10–silica composites showing the strongest and most homogeneous contrast of otherwise non-stained samples. This template was therefore selected for a twelve day time course experiment to investigate the growth kinetics of its mineral shells, and if the thickness of the silica coating might be controlled via the TEOS incubation time. Total widths of randomly selected, low-contrast TMV–KD10 cores surrounded by electron-dense sheaths were measured in digital TEM images by help of image processing software from the fifth day onwards. This revealed an increase of layer thickness with progressing time (Figure 5). After ten days of reaction time, the TMV–silica hybrids exhibited average diameters of about 29 ± 2 nm, which did not further increase upon extended incubation. At the same time, granular SiO_2_ deposits began to differentiate on the nanotube surfaces, rendering them less smooth than during earlier stages. The overall diameter, that is, the height of TMV–KD10-templated hybrid rods, after ten days of mineralization was additionally measured by AFM (data not shown). For this purpose, mineralized viruses were deposited on a silicon substrate. The average of the resulting mean values of the virus height was in good agreement with the TEM data and revealed a typical particle diameter of 30 nm, corresponding to a ≈6 nm linker–peptide–silica coating of the 18 nm TMV core. Different from non-modified viral rods immobilized on a silicon substrate, where reduction of the virus height due to attraction to the substrate surface is observed [32], the adhesion of mineralized viruses from suspensions to the wafer substrates did not reduce the objects' height. This indicated the formation of a rigid composite not radially compressed upon its surface adsorption.

### ToF-SIMS analysis of the deposited material

**Figure 6:**
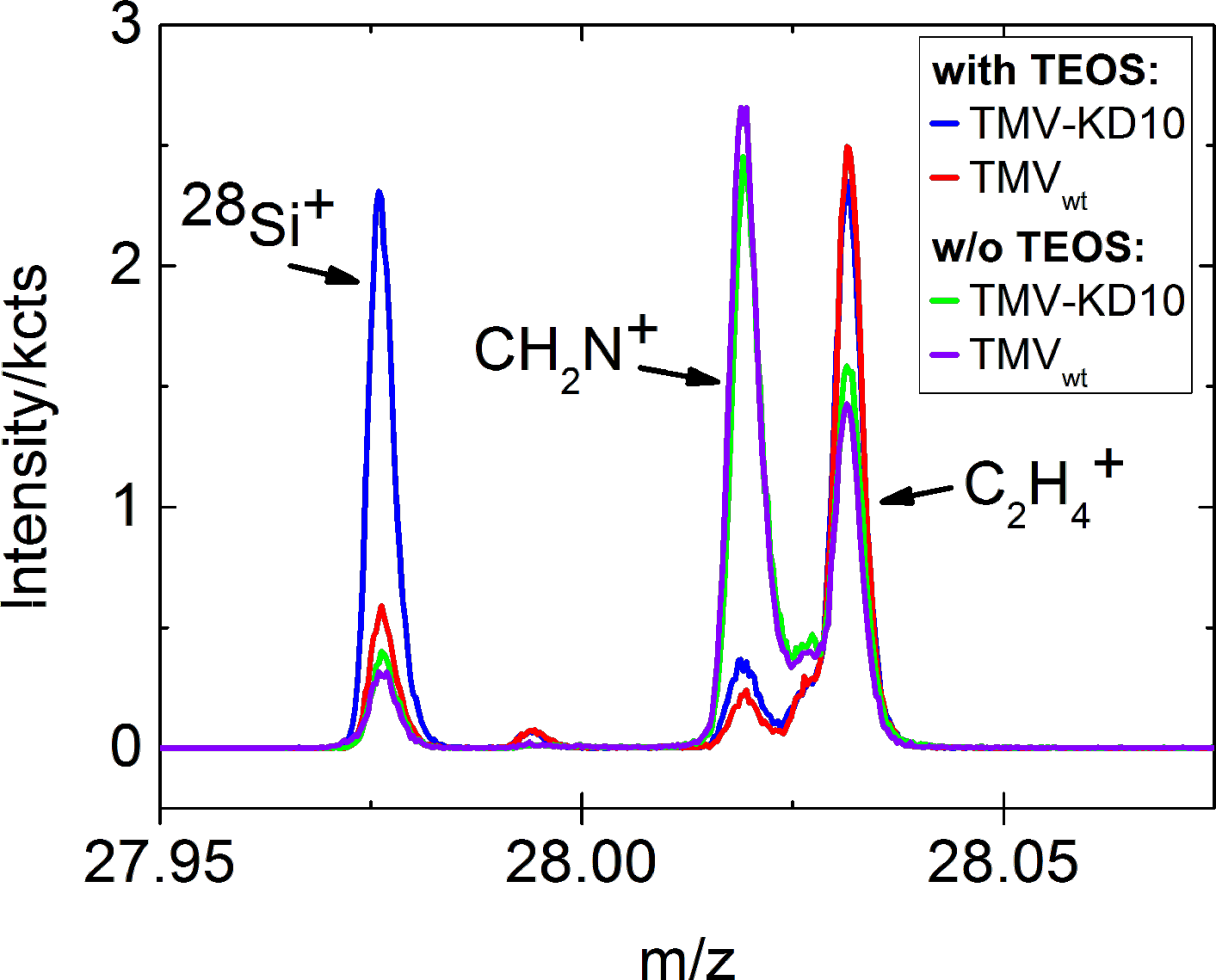
ToF-SIMS analysis for determination of silica deposition. TMV–KD10 with TEOS (blue) and without TEOS (green), TMV_wt_ with TEOS (red) and without TEOS (purple) after ten days of incubation. The peak at *m*/*z* 27.97 indicates Si, the peak at m/z 28.02 CH_2_N^+^, and the peak at m/z 28.03 C_2_H_4_^+^. For TMV–KD10 with TEOS and TMV_wt_ with TEOS, the decrease of the CH_2_N^+^ peak, indicating peptide/protein components, is an indirect effect of the mineralization, shielding the soft-matter surface of biotemplate particles.

An analysis of the deposited materials with time-of-flight secondary ion mass spectrometry (ToF-SIMS) [90] was performed on air-dried, drop cast suspensions of TMV_wt_ or TMV–KD10 particles (both with and without 10 days of exposure to TEOS). Positive and negative secondary ion spectra were recorded from random positions of the TMV deposits. The peak assignment is based on high mass resolution data and isotope patterns for Si. As shown in Figure 6, the intensity of the Si^+^ signal decreases substantially from TMV–KD10 particles incubated with TEOS (blue) to the TMV_wt_ control with TEOS (red) to both negative controls not incubated in TEOS solutions (green and purple). Analyzing SiOH^+^ and several fragments characteristic of silica in negative polarity spectra (SiO_2_^−^, SiO_3_^−^, SiO_3_H^−^) indicated the same trend. Since the sample preparation method did not yield fully TMV-covered samples, the recorded mass spectra averaged over a field of view of 500 × 500 µm^2^ show individual levels of Au^−^ stemming from the underlying substrate. In order to correct for this dispersion or area effect, the raw intensities of Si^+^ and SiOH^+^ were normalized according to the gold signals of each analyzed spot. Semi-quantitative silicification levels obtained thereof are presented in Table 3.

**Table 3:** Relative silicification levels determined from normalized Si^+^ and SiOH^+^ intensities in SIMS. TEOS exposure 10 days, when applicable.

Construct	Normalized Si^+^	Normalized SiOH^+^

TMV–KD10 with TEOS	100%	100%
TMV_wt_ with TEOS	18%	17%
TMV–KD10 without TEOS	1%	1%
TMV_wt_ without TEOS	<1%	<1%

As shown in Table 3, the normalized intensities of two silica-derived fragments obtained with SIMS allow for a rough but reasonable quantification of the conversion of TEOS to silica induced by bare and KD10-functionalized TMV particles. While all negative controls not exposed to TEOS show negligible levels of Si^+^ and SiOH^+^, both TMV–KD10 and TMV_wt_ exposed to TEOS did form insoluble silica to considerably different extents. This resulted in about five times higher amounts of mineral on the peptide-modified virus. This finding is in agreement with our microscopic observations, which were not sufficient to resolve the deposition of silica on the wild type viral template. The ToF-SIMS analysis therefore revealed either a spontaneous hydrolysis of TEOS (also occurring in the absence of effector peptides) or a low but specific mineralization-promoting activity of the bare viral CP surface (not detectable by electron-optical imaging). The low SIMS Si^+^ and SiOH^+^ signal intensities, and the necessary high mass resolution for unambiguous fragment assignments, precluded SIMS imaging with high lateral resolution. Hence, the obtained SIMS data cannot visualize mineralized individual TMV particles or distinguish between silica bound to virus particles and silica deposited by self-hydrolysis.

## Conclusion

A systematic comparison of TMV-based nanobiotemplates chemically functionalized with different types of mineralization-affecting peptides revealed superior capacities of repetitive, alternating KD sequences in guiding the deposition of silica sheaths from TEOS precursor solutions around the viral soft-matter cores. The peptide KD10 designed in this study on the basis of earlier tests [61] allowed for the most selective and controllable silicification by sol–gel condensation. This was likely due to its charge-relay activity, in comparison to different histidine-containing effector peptides and the bare or linker-coated viral scaffold surface. To our knowledge, this is the first evaluation of peptide-equipped TMV templates with regard to the generation of silica nanostructures of adjustable diameter. The previous studies of other researchers, all of which employed natural or aniline-modified TMV to nucleate silica deposition (as specified above), yielded either nanometric coatings of individual particles, or differently organized bio-inorganic mesostructures, but did not focus on fine-tuning the growth of the silica shells on the one-to-ten nm range. This was intended here and best achieved by the KD10-exposing TMV variant, for which a convincing correlation between silicification time and mineral layer thickness could be demonstrated.

The KD10-fashioned plant viruses thus enable the one-pot manufacture of freely suspended silica nanorods with a soft-matter core, devoid of significant amounts of byproducts. It would be interesting to characterize the mechanical properties of these composites in comparison to synthetically synthesized silica nanorods. This could potentially lead to fundamentally novel types of fiber-reinforced biohybrid materials. Furthermore, the method may also give rise to an efficient fabrication of rigid, ultrasmall components of unusual shapes, on the basis of different nonlinear kinked and branched TMV-based architectures generated recently in our lab [54].

Finally, peptides spatially immobilized in a selective manner on certain target sites of biotemplates might also be a clue to the use of silica deposition as a “bionic glue”. On appropriate TMV variants, specific coupling groups of amino acids are confined to outer, inner or end surfaces of the nucleoprotein tubes, respectively. Serial in vitro assembly of different genetically engineered CP types on RNA scaffolds can even generate nanorod subdomains, offering unique coupling functionality [76]. Addressing such sites for a selective conjugation of mineralization-guiding peptides such as KD10 might pave future routes towards a firm and controlled integration of TMV-based nanostructures into miniaturized devices. Here they might act, for example, as adaptor templates, enabling an ultradense presentation of functional molecules on the non-mineralized regions of their multivalent protein surfaces.

Taken together, extended composite bio-hybrid materials and complex miniaturized systems both might profit from the precise shapes, high availabilities and immense in vitro tuning potential of plant viral templates, and their peptide-controlled transformation into mineralized nanostructured composites adapted to specific future applications.

## Experimental

### Materials

The peptides, (KD)_5_C, (KD)_10_C, and CA_4_H_4_, of 90% purity were provided by GeneCust (Dudelange, Luxembourg). Peptides 31C and 44C were purchased from EMC Microcollections (Tübingen, Germany).

### TMV functionalization with bifunctional linker molecules and peptides

Wild type TMV_wt_ and genetically modified TMV_Lys_ [76] (also named TMV–Lys here to underscore the functional amino groups exposed by its lysine side chains) were purified according to Gooding and Hebert [91]. Peptide conjugation onto the virus surface followed a protocol established on the basis of literature data [92–93] and instruction kindly provided by Sourabh Shukla and Nicole Steinmetz, Case Western Reserve University, Cleveland, Ohio, U.S.A. For this procedure, 1200 µL of TMV–Lys particles (5 mg/mL) in 10 mM sodium potassium phosphate (SPP) pH 7.2 were mixed with 9 µL of 1 M hetero-bifunctional crosslinker, succinimidyl-(*N*-maleimidopropionamido)-tetraethylene glycol ester (SM(PEG)_4_, Thermo Scientific, Karlsruhe, Germany) dissolved in dimethyl sulfoxide and incubated at 37 °C for 2 h under agitation (horizontal shaking at 500 rpm). The TMV particles were sedimented for 1.5 h at 90,500 *g* and 4 °C in an Optima L-90K ultracentrifuge (Beckman Coulter, Krefeld, Germany). The resulting pellet of linker-equipped TMV (named TMV–PEG) was resuspended in 600 µL of 10 mM SPP pH 7.2. A volume of 100 µL of TMV–PEG solution was mixed with 800 µL of 10 mM SPP pH 7.2 and 40 µL of peptides (3.3 mg/mL) dissolved in dimethylformamide and incubated at 30 °C for 2 h and subsequently at 4 °C overnight under agitation as above. The TMV particles with conjugated peptides were sedimented by ultracentrifugation as above. The pellets were washed with 1 mL of ultrapure water (ddH_2_O, 18.3 MΩ cm, purified by a membraPure system, Aquintus, Bodenheim, Germany) and resuspended in 100 µL of ddH_2_O. The TMV_wt_ and TMV–Lys concentrations were determined by UV spectroscopy with a NanoDrop ND-1000 spectrophotometer (PeqLab, Erlangen, Germany) at a wavelength of 260 nm, using the extinction coefficient of TMV particles (3 mL mg^−1^ cm^−1^ [94]) . For estimating concentrations of the different biotemplate rods, the band intensities of modified CPs and unmodified CP_Lys_ after SDS-PAGE separation and Comassie Blue staining were compared (see below).

### Electrophoretic analysis

The modified CPs were analyzed by denaturing SDS-PAGE [95]. Samples containing 0.2 µg of protein were heated for 5 min at 95 °C in sample buffer (50 mM Tris-HCl (tris-(hydroxymethyl)aminomethan hydrochloride) pH 6.8, 2% (w/v) SDS, 0.1% (w/v) bromophenol blue, 10% glycerol, 100 mM dithiothreitol) and separated on 15% PA gels. Fixed gels were stained with Coomassie Brilliant Blue R250 (Serva Electrophoresis, Heidelberg, Germany) according to standard procedures [96].

Modified and unmodified TMV–Lys templates were separated as intact particles in native 0.9% agarose gels in 98 mM Tris pH 8.0, 89 mM boric acid, 2 mM EDTA. 12 µg of total protein in sample buffer (10 mM SPP pH 7.2, 0.1% (w/v) bromophenol blue, 10% glycerol) were applied per lane. TMV bands were stained with Coomassie Brilliant Blue R250.

### Zeta potential determination and charge calculation

The zeta potential was measured with a Malvern Zetasizer Nano ZS (Malvern Instruments, Worcestershire, UK) using disposable folded cuvettes. The Smoluchowski approximation was used according to instrument settings to convert the electrophoretic mobility to a zeta potential. The experiments consisted of 30 runs per measurement. All experiments were conducted in triplicate. The zeta potential was measured for each sample with a concentration of 0.5 mg/mL TMV particles solution in ddH_2_O (pH 5.5) as well as in 30 mM Tris-HCl at pH 8.0.

### TMV particle mineralization

Peptide-functionalized TMV templates resuspended in water (see above) were kept for one to two days at 4 °C to allow their complete dispersion after ultracentrifugation. For the mineralization of particles with and without linkers and conjugated peptides, a 40 µL TMV template solution (10 mg/mL) was mixed with 50 µL 20% (v/v) TEOS (Sigma-Aldrich, München, Germany) in ethanol (99.8% p.a.), resulting in final concentrations in the mineralization reaction mixture of 4.4 mg/mL TMV, 11.1% (v/v) TEOS, and 44.4% (v/v) ethanol in an aqueous solution of pH 5.5–5.6. It was crucial to mix TEOS and ethanol before combining it with TMV particles in order to preserve their structural integrity. Mineralization reactions were incubated for 1, 2, 5, 7, 10 or 12 days under agitation (horizontal shaking at 500 rpm) at 25 °C. The reaction mixture was precipitated in a table centrifuge for 15 min at 20,000*g* and 18 °C. The supernatant was discarded and the pellet washed twice with 200 µL of 50% (v/v) ethanol to remove residual unconverted TEOS. The pellet was resuspended in 50 µL of ddH_2_O and centrifuged for 30 min at 10,000*g*. The resulting pellet was dissolved in 50 µL of ultrapure water.

### Characterization of mineralized TMV particles

The surface of mineralized TMV particles was characterized by SEM analysis. 20 µL of 1:250 diluted, mineralized TMV solutions in ultrapure water (for the mineralized TMV particle solution preparation see the previous section) were pipetted on n-Si wafer substrates and air dried. The samples were analyzed in an ultrahigh resolution field emission SEM (FE-SEM; S-5200, Hitachi Ltd., Tokyo, Japan) at 30 kV.

The TEM analysis was carried out to determine the silica shell thickness of TMV–KD10 particles after different reaction times. 3 µL of mineralized TMV particles in solution were incubated on a 400-mesh formvar, carbon-covered copper grid for 5 min. The droplet was removed with five droplets of ultrapure water and air dried. The samples were analyzed under a Zeiss EM-10A TEM (Carl Zeiss, Oberkochen, Germany) at 60 kV.

For ToF-SIMS analysis, Si chips (5 × 10 mm) were cut from n-Si wafers (CrysTec, Berlin, Germany) and used as supporting substrates. These were coated with a 4 nm thick chromium layer for adhesion and a 30 nm thick gold layer by physical vapor deposition (PVD; Varian NRC 836, Palo Alto, California, U.S.A.). All samples used for mineralization analysis were found to be free of Si and silicon oil contamination, which could potentially interfere with the analysis.

10 µL of a 1:250 diluted solution of mineralized TMV or control preparations in ultrapure water (see TMV particle mineralization) were pipetted on a gold-covered n-Si wafer and air dried. ToF-SIMS was performed on a TOF.SIMS5 instrument (ION-TOF GmbH, Münster, Germany). The spectrometer was equipped with a Bi cluster primary ion source and a reflection-type time-of-flight analyzer. The UHV base pressure was <5 × 10^−9^ mbar. For high mass resolution, the Bi source was operated in the “high current bunched” mode, providing short Bi_1_^+^ primary ion pulses at 25 keV energy and a lateral resolution of approximately 4 μm. The short pulse length of 0.6 to 1.0 ns allowed for high mass resolution. The primary ion beam was rastered across a 500 × 500 µm^2^ field of view on the sample, and 128 × 128 data points were recorded. Primary ion doses were kept below 10^11^ ions/cm^2^ (static SIMS limit). The spectra were calibrated against C^−^, CH^−^, CH_2_^−^, and Au^-^, or on the C^+^, CH^+^, CH_2_^+^, and CH_3_^+^ peaks, respectively. Based on these datasets, the chemical assignments for characteristic fragments were determined.

## References

[1] Leblanc J L (2002). Prog Polym Sci.

[2] Bouwmeester H, Brandhoff P, Marvin H J P, Weigel S, Peters R J B (2014). Trends Food Sci Technol.

[3] Stark W J, Stoessel P R, Wohlleben W, Hafner A (2015). Chem Soc Rev.

[4] Henstock J R, Canham L T, Anderson S I (2015). Acta Biomater.

[5] Vallet-Regí M, Ruiz-Hernández E (2011). Adv Mater.

[6] Tang F, Li L, Chen D (2012). Adv Mater.

[7] Yildirim A, Acar H, Erkal T S, Bayindir M, Guler M O (2011). ACS Appl Mater Interfaces.

[8] Yang X, Tang H, Cao K, Song H, Sheng W, Wu Q (2011). J Mater Chem.

[9] Mann S (2009). Nat Mater.

[10] Paris O, Burgert I, Fratzl P (2010). MRS Bull.

[11] Zollfrank C, Scheibel T, Seitz H, Travitzky N (2014). Bioinspired Materials Engineering. Ullmann's Encyclopedia of Industrial Chemistry.

[12] Mann S (1983). Mineralization in biological systems. Inorganic Elements in Biochemistry.

[13] Weiner S, Dove P M (2003). Rev Mineral Geochem.

[14] Wu Z J, Xiang H, Kim T, Chun M-S, Lee K (2006). J Colloid Interface Sci.

[15] Cademartiri R, Brook M A, Pelton R, Brennan J D (2009). J Mater Chem.

[16] Aljabali A A A, Shah S N, Evans-Gowing R, Lomonossoff G P, Evans D J (2011). Integr Biol.

[17] Yuwono V M, Hartgerink J D (2007). Langmuir.

[18] Bittner A M, Alonso J M, Górzny M Ł, Wege C, Mateu M G (2013). Nanoscale Science and Technology with Plant Viruses and Bacteriophages. Structure and physics of viruses: An integrated textbook.

[19] Mao C, Liu A, Cao B (2009). Angew Chem, Int Ed.

[20] Lomonossoff G P, Evans D J, Palmer K, Gleba Y (2014). Applications of Plant Viruses in Bionanotechnology. Plant Viral Vectors.

[21] Shenton W, Douglas T, Young M, Stubbs G, Mann S (1999). Adv Mater.

[22] Pouget E, Grelet E (2013). Langmuir.

[23] Yang S H, Chung W-J, McFarland S, Lee S-W (2013). Chem Rec.

[24] Li D, Qu X, Newton S M C, Klebba P E, Mao C (2012). J Mater Chem.

[25] Niu Z, Liu J, Lee L A, Bruckman M A, Zhao D, Koley G, Wang Q (2007). Nano Lett.

[26] Royston E S, Brown A D, Harris M T, Culver J N (2009). J Colloid Interface Sci.

[27] Aljabali A A A, Barclay J E, Cespedes O, Rashid A, Staniland S S, Lomonossoff G P, Evans D J (2011). Adv Funct Mater.

[28] Rothenstein D, Facey S J, Ploss M, Hans P, Melcher M, Srot V, Van Aken P A, Hauer B, Bill J (2013). Bioinspired, Biomimetic Nanobiomater.

[29] Evans D J (2009). Biochem Soc Trans.

[30] Dujardin E, Peet C, Stubbs G, Culver J N, Mann S (2003). Nano Lett.

[31] Yang C, Choi C-H, Lee C-S, Yi H (2013). ACS Nano.

[32] Knez M, Sumser M P, Bittner A M, Wege C, Jeske H, Hoffmann D M P, Kuhnke K, Kern K (2004). Langmuir.

[33] Fujikawa S, Kunitake T (2003). Langmuir.

[34] Royston E, Lee S-Y, Culver J N, Harris M T (2006). J Colloid Interface Sci.

[35] Chiang C-Y, Epstein J, Brown A, Munday J N, Culver J N, Ehrman S (2012). Nano Lett.

[36] Mao C, Solis D J, Reiss B D, Kottmann S T, Sweeney R Y, Hayhurst A, Georgiou G, Iverson B, Belcher A M (2004). Science.

[37] Atanasova P, Rothenstein D, Schneider J J, Hoffmann R C, Dilfer S, Eiben S, Wege C, Jeske H, Bill J (2011). Adv Mater.

[38] Balci S, Bittner A M, Schirra M, Thonke K, Sauer R, Hahn K, Kadri A, Wege C, Jeske H, Kern K (2009). Electrochim Acta.

[39] Atanasova P, Stitz N, Sanctis S, Maurer J H M, Hoffmann R C, Eiben S, Jeske H, Schneider J J, Bill J (2015). Langmuir.

[40] Adams M J, Heinze C, Jackson A O, Kreuze J F, MacFarlane S A, Torrance L, King A M Q, Adams M J, Carstens E B (2012). Family Virgaviridae. Virus taxonomy: Ninth report of the International Committee on Taxonomy of Viruses.

[41] Culver J N (2002). Annu Rev Phytopathol.

[42] Butler P J G (1999). Philos Trans R Soc, B.

[43] Namba K, Pattanayek R, Stubbs G (1989). J Mol Biol.

[44] Ge P, Zhou Z H (2011). Proc Natl Acad Sci U S A.

[45] Clare D K, Orlova E V (2010). J Struct Biol.

[46] Sachse C, Chen J Z, Coureux P D, Stroupe M E, Fändrich M, Grigorieff N (2007). J Mol Biol.

[47] Alonso J M, Górzny M Ł, Bittner A M (2013). Trends Biotechnol.

[48] Culver J N, Brown A D, Zang F, Gnerlich M, Gerasopoulos K, Ghodssi R (2015). Virology.

[49] Schlick T L, Ding Z, Kovacs E W, Francis M B (2005). J Am Chem Soc.

[50] Smith M L, Lindbo J A, Dillard-Telm S, Brosio P M, Lasnik A B, McCormick A A, Nguyen L V, Palmer K E (2006). Virology.

[51] Eiben S, Stitz N, Eber F, Wagner J, Atanasova P, Bill J, Wege C, Jeske H (2014). Virus Res.

[52] Shukla S, Eber F, Nagarajan A S, DiFranco N A, Schmidt N, Wen A M, Eiben S, Twyman R M, Wege C, Steinmetz N F (2015). Adv Healthcare Mater.

[53] Eber F J, Eiben S, Jeske H, Wege C (2013). Angew Chem, Int Ed.

[54] Eber F J, Eiben S, Jeske H, Wege C (2015). Nanoscale.

[55] Baio J E, Zane A, Jaeger V, Roehrich A M, Lutz H, Pfaendtner J, Drobny G P, Weidner T (2014). J Am Chem Soc.

[56] Zane A C, Michelet C, Roehrich A, Emani P S, Drobny G P (2014). Langmuir.

[57] Cha J N, Shimizu K, Zhou Y, Christiansen S C, Chmelka B F, Stucky G D, Morse D E (1999). Proc Natl Acad Sci U S A.

[58] Kröger N, Deutzmann R, Sumper M (1999). Science.

[59] Poulsen N, Kröger N (2004). J Biol Chem.

[60] Rothenstein D, Claasen B, Omiecienski B, Lammel P, Bill J (2012). J Am Chem Soc.

[61] Kuno T, Nonoyama T, Hirao K, Kato K (2011). Langmuir.

[62] Stöber W, Fink A, Bohn E (1968). J Colloid Interface Sci.

[63] Kim Y J, Hwang K H, Park S-J, Jeon D-Y, Nam C-H, Kim G-T (2013). J Nanosci Nanotechnol.

[64] Fowler C E, Shenton W, Stubbs G, Mann S (2001). Adv Mater.

[65] Rong J, Oberbeck F, Wang X, Li X, Oxsher J, Niu Z, Wang Q (2009). J Mater Chem.

[66] Mueller A, Eber F J, Azucena C, Petershans A, Bittner A M, Gliemann H, Jeske H, Wege C (2011). ACS Nano.

[67] Chen X, Gerasopoulos K, Guo J, Brown A, Ghodssi R, Culver J N, Wang C (2011). Electrochim Acta.

[68] Li F, Wang Q (2014). Small.

[69] Luckanagul J, Andrew Lee L, Nguyen Q L, Sitasuwan P, Yang X, Shazly T, Wang Q (2012). Biomacromolecules.

[70] Wu Z, Mueller A, Degenhard S, Ruff S E, Geiger F, Bittner A, Wege C, Krill C E (2010). ACS Nano.

[71] Luckanagul J A, Lee L A, You S, Yang X, Wang Q (2015). J Biomed Mater Res, Part A.

[72] Patwardhan S V, Emami F S, Berry R J, Jones S E, Naik R R, Deschaume O, Heinz H, Perry C C (2012). J Am Chem Soc.

[73] Steinmetz N F, Shah S N, Barclay J E, Rallapalli G, Lomonossoff G P, Evans D J (2009). Small.

[74] Gebauer D, Kellermeier M, Gale J D, Bergström L, Cölfen H (2014). Chem Soc Rev.

[75] Almora-Barrios N, Austen K F, de Leeuw N H (2009). Langmuir.

[76] Geiger F C, Eber F J, Eiben S, Mueller A, Jeske H, Spatz J P, Wege C (2013). Nanoscale.

[77] (2015). Protein Calculator V3.4.

[78] (2011). ImageJ software.

[79] Hong J H, Duncan S E, Dietrich A M (2010). Food Qual Prefer.

[80] Hunter R J (1981). Zeta potential in colloid science: principles and applications.

[81] Müller R H, Hildebrand G E (1995). Zetapotential und Partikelladung in der Laborpraxis: Einführung in die Theorie praktische Messdurchführung Dateninterpretation.

[82] Reed J S (1995). Principles of ceramics processing.

[83] Niu Z, Kabisatpathy S, He J, Lee L A, Rong J, Yang L, Sikha G, Popov B, Emrick T, Russell T (2009). Nano Res.

[84] Bruckman M A, Soto C M, McDowell H, Liu J L, Ratna B R, Korpany K V, Zahr O K, Blum A S (2011). ACS Nano.

[85] Kadri A, Maiß E, Amsharov N, Bittner A M, Balci S, Kern K, Jeske H, Wege C (2011). Virus Res.

[86] Altunbas A, Sharma N, Lamm M S, Yan C Q, Nagarkar R P, Schneider J P, Pochan D J (2010). ACS Nano.

[87] Acar H, Garifullin R, Guler M O (2011). Langmuir.

[88] Sumper M, Kröger N (2004). J Mater Chem.

[89] Filner B, Marcus A (1974). Virology.

[90] Belu A M, Graham D J, Castner D G (2003). Biomaterials.

[91] Gooding G V, Hebert T T (1967). Phytopathology.

[92] Bruckmann M A, Steinmetz N F, Lin B, Ratna B (2014). Chemical Modification of the Inner and Outer Surfaces of Tobacco Mosaic Virus (TMV). Virus hybrids as nanomaterials: methods and protocols.

[93] Bruckman M A, Jiang K, Simpson E J, Randolph L N, Luyt L G, Yu X, Steinmetz N F (2014). Nano Lett.

[94] (2015). Description of Plant Viruses.

[95] Laemmli U K (1970). Nature.

[96] Green M R, Sambrook J (2012). Molecular cloning: a laboratory manual.

